# Additional value of cardiac magnetic resonance feature tracking parameters for the evaluation of the arrhythmic risk in patients with mitral valve prolapse

**DOI:** 10.1186/s12968-023-00944-x

**Published:** 2023-06-15

**Authors:** Marco Guglielmo, Dimitri Arangalage, Marco Augusto Bonino, Gianmarco Angelini, Michela Bonanni, Gianluca Pontone, Patrizio Pascale, Laura Anna Leo, Francesco Faletra, Jurg Schwitter, Giovanni Pedrazzini, Pierre Monney, Anna Giulia Pavon

**Affiliations:** 1 Division of Heart and Lungs, Department of Cardiology, Utrecht University, Utrecht University Medical Center, Utrecht, The Netherlands; 2 Department of Cardiology, Haga Teaching Hospital, The Hague, The Netherlands; 3 Cardiology Department, AP-HP, Bichat Hospital and Université de Paris, Paris, France; 4 Department of Surgery, Hopital Universitaire Genève, Geneva, Switzerland; 5 Cardiology Unit, Department of Emergency and Organ Transplantation, University of Bari Aldo Moro, Policlinico of Bari, University Hospital, Bari, Italy; 6 Department of Experimental Medicine, University of Rome “Tor Vergata”, Rome, Italy; 7 Centro Cardiologico Monzino IRCCS, Milan, Italy; 8 Division of Cardiology, Cardiocentro Ticino Institute, Ente Ospedaliero Cantonale, Via Tesserete, 48, 6900 Lugano, Switzerland; 9 Department of Cardiology, Lausanne University Hospital (CHUV), Lausanne, Switzerland; 10 Center for Cardiac Magnetic Resonance of the CHUV (CRMC), Lausanne University Hospital, Lausanne, Switzerland; 11 University of Lausanne (UniL), Lausanne, Switzerland

**Keywords:** Mitral valve prolapse, Cardiovascular magnetic resonance, Mitral annular disjunction, Interstitial fibrosis, Strain

## Abstract

**Objectives:**

The identification of patients with mitral valve prolapse (MVP) presenting high arrhythmic risk remains challenging. Cardiovascular Magnetic Resonance (CMR) feature tracking (FT) may improve risk stratification. We analyzed the role of CMR-FT parameters in relation to the incidence of complex ventricular arrhythmias (cVA) in patients with MVP and mitral annular disjunction (MAD).

**Methods:**

42 patients with MVP and MAD who underwent 1.5 T CMR were classified as MAD-cVA (n = 23, 55%) in case of cVA diagnosed on a 24-h Holter monitoring and as MAD-noVA in the absence of cVA (n = 19, 45%). MAD length, late gadolinium enhancement (LGE), basal segments myocardial extracellular volume (ECV) and CMR-FT were assessed.

**Results:**

LGE was more frequent in the MAD-cVA group in comparison with the MAD-noVA group (78% vs 42%, p = 0.002) while no difference was observed in terms of basal ECV. Global longitudinal strain (GLS) was reduced in MAD-cVA compared to MAD-noVA (− 18.2% ± 4.6% vs − 25.1% ± 3.1%, p = 0.004) as well as global circumferential strain (GCS) at the mid-ventricular level (− 17.5% ± 4.7% vs − 21.6% ± 3.1%, p = 0.041). Univariate analysis identified as predictors of the incidence of cVA: GCS, circumferential strain (CS) in the basal and mid infero-lateral wall, GLS, regional longitudinal strain (LS) in the basal and mid-ventricular inferolateral wall. Reduced GLS [Odd ratio (OR):1.56 (confidence interval (CI) 95%: 1.45–2.47; p < 0.001)] and regional LS in the basal inferolateral wall [OR: 1.62 (CI 95%: 1.22–2.13; p < 0.001)] remained independent prognostic factors in multivariate analysis.

**Conclusion:**

In patients with MVP and MAD, CMR-FT parameters are correlated with the incidence of cVA and may be of interest in arrhythmic risk stratification.

**Supplementary Information:**

The online version contains supplementary material available at 10.1186/s12968-023-00944-x.

## Introduction

Mitral valve prolapse (MVP) is the most frequent cause of primary mitral regurgitation (MR) in Western countries with an estimated prevalence of 2%. Despite its overall good prognosis [1–3] since its first description, it has been associated with ventricular arrhythmia (VA), including sustained ventricular tachycardia (SVT), ventricular fibrillation (VF) and sudden cardiac death (SCD) [4]. Cardiac imaging plays a key role in the diagnosis of MVP. The presence of a bileaflet MVP, together with mitral annular disjunction (MAD) and myocardial fibrosis detected on LGE are considered features linked to a high risk of arrhythmia [5]. However, the nature of this relationship remains only partially deciphered and identifying prognostic factors and mechanisms associated with these events is currently emerging as a challenging task [6, 7]. Basso et al. histologically demonstrated in a landmark publication the correlation between SCD and fibrosis of the papillary muscles and inferobasal left ventricular (LV) wall [8]. These findings were confirmed in a cohort of living patients affected by both MVP and VA, in which LV late gadolinium enhancement (LGE) was identified by contrast-enhanced cardiac magnetic resonance (CMR) in 93% of patients [8]. Moreover, there is evidence in the literature supporting an association between MAD, LV inferobasal LGE and VA [9]. Beyond the increasingly well-known association between MAD, the presence of macroscopic fibrosis of the papillary muscles and LV inferolateral wall detected by LGE and VA, recent findings have highlighted a possible role of interstitial fibrosis [10, 11]. In patients with MVP and MAD, diffuse interstitial fibrosis was detected by T1 mapping even in the absence of macroscopic fibrosis on LGE sequences and seems to provide additional value beyond the assessment of LGE in the arrhythmic risk stratification. Several patterns of LV remodeling may therefore be linked to the arrhythmic risk: either focal replacement fibrosis assessed by LGE or interstitial fibrosis measured with T1 mapping [10].

CMR feature tracking (CMR-FT) is emerging as a potential tool for early detection of subtle morphologic and functional alterations in various cardiac conditions since it is able to identify LV deformation abnormalities even in the presence of normal parameters of systolic function. Subtle myocardial deformation changes have been associated with tissue changes in MVP patients detected by native T1 [11]. To date, data on the role of CMR-FT and T1-mapping in patients with MVP and MAD have seldom been reported. The aim of the present study is to assess the additional value of CMR-FT and T1 mapping in the arrhythmic risk assessment of patients with MVP and MAD.

## Methods

### Study population

All consecutive patients with a bileaflet MVP and MAD referred to CMR between January 2016 and July 2021 in the setting of risk assessment were retrospectively included in the present study. Inclusion criteria were age > 18 years old and the presence of isolated bileaflet MVP. MVP was defined as a systolic excursion of the mitral leaflets > 2 mm behind the mitral annular plane in the long axis view, i.e. a displacement of > 2 mm into the left atrium (LA). MAD was defined as an anatomic variant of the posterior mitral annulus resulting in an abnormal separation (≥ 2.0 mm) between the left atrial wall/mitral valve junction and LV inferolateral wall on the cine 3-chamber end-systolic image, as previously reported in literature [7–9] (Additional file 1: Fig. S1). Exclusion criteria were secondary mitral regurgitation, history of myocardial infarction, myocarditis, hypertrophic cardiomyopathy, infiltrative heart disease, more than mild associated other valvular heart disease and/or a LV ejection fraction (LVEF) < 50%, atrial fibrillation, presence of implantable cardioverter defibrillator or pacemaker. A group of 20 subjects who underwent a CMR examination for other suspected medical conditions but with normal findings formed the control group. All these 20 subjects underwent a transthoracic echocardiography prior to CMR that excluded the presence of MVP.

The study complied with the declaration of Helsinki and the ethics committee approved the research protocol. All patients provided written informed consent for their inclusion.

### Cardiovascular magnetic resonance protocol

ECG-gated CMR imaging was performed using a 1.5 T magnet (Siemens Healthcare, MAGNETOM Aera or Sola, Erlangen, Germany) with a 32-channel phased-array surface receiver coil. Cine images were acquired using a breath-hold steady-state free precession (SSFP) sequence in long-axis (2-chamber, 3-chamber and 4-chamber) and short-axis orientations (8 mm slices without gap, 10–15 slices).

Ten minutes after the administration of a 0.2 mmol/kg intravenous bolus of Gadobutrol (Gadovist, Bayer Healthcare, Berlin, Germany) at a flow rate of 4 mL/s followed by saline flush/ LGE images were acquired using a 2D breath-hold phase-sensitive segmented inversion-recovery gradient echo pulse sequence in the same orientations as cine images. The following parameters were used: FOV: 380 to 420 mm; TR/TE: 4.6/1.3 ms; α: 20°; matrix: 256 × 192; slice thickness: 8 mm; no interslice gap. Inversion time was individually optimized to null normal myocardium (usual range 220 to 300 ms).

For pre-contrast T1 mapping, we used the ECG-triggered modified Look-Locker inversion recovery (MOLLI) sequence (using the scheme 3 (3)3(3)5) on a single short-axis basal LV slice. Post-contrast T1-mapping was acquired following LGE imaging (20 min after Gadobutrol bolus injection) using a MOLLI sequence (4(1)3(1)2 scheme). The basal slice was defined as the slice position of the most basal cine SSFP slice, where a complete ring of myocardium was visible throughout diastole and systole.

MR severity was assessed by phase contrast flow images. MR volume was calculated according to the standard indirect method, by subtracting the forward aortic flow volume from the total LV stroke volume (LV stroke volume − forward aortic flow). The MR regurgitant fraction was quantified using the following equation: (regurgitant volume × 100)/(LV stroke volume). According to the literature MR was graded as mild if MR regurgitant fraction was < 20%, moderate if 20–39% and severe if > 40% [12].

### Image analysis

All CMR examinations were analyzed blinded to clinical characteristics and outcome using the Syngo.via software (Siemens Healthineers, Erlangen, Germany) to calculate LV volumes, LV mass and LVEF by delineating endocardial and epicardial borders in the stack of short-axis cine images. MAD was assessed on a 3-chamber view in systole and defined as any detachment of the MV leaflet junction from the top of the basal LV inferolateral wall to the left atrial wall [8, 9].

The presence of myocardial LGE was defined as hyperintense myocardium with a signal intensity > 5 SDs above the mean signal intensity of normal myocardium. The extent of LGE was semi-quantitatively reported according to the American Heart Association 17 segments model [13].

Pre- and post-contrast T1 mapping images were obtained in the basal slice and first visually reviewed to assess quality. Myocardial T1 relaxation times and ECV were then measured in every myocardial segment according to AHA classification and divided in anterior, anteroseptal, inferoseptal, inferior, inferolateral and anterolateral wall as previously reported in literature [10].

CMR-FT analysis was performed using commercially available software (QStrain, Medis medical Imaging system version 3.2.60.6, Leiden, The Netherlands). Long-axis SSFP cine images (4-chamber, 3-chamber and 2-chamber) were used to derive global longitudinal strain (GLS). Segments with poor tracking were excluded from further analysis and if more than two segments demonstrated poor tracking, the view was excluded from further analysis. GLS values were obtained by averaging the segmental strain values (6 segments in each of the 4-chamber, 2-chamber and 3-chamber views for a total of 18 segments when data from all segments were available). Global circumferential strain (GCS) was evaluated considering the basal slice, mid-ventricular slice, at the level of papillary muscle, and apical slice. Regional longitudinal strain (LS) and regional circumferential strain (CS) were also assessed. To assess regional LS strain values for the six basal, six mid, and six apical segments of the LV were analyzed separately as antero-lateral, infero-lateral, inferior, infero-septal and antero-septal wall at basal, mid-ventricular, and apical level and successively averaged to obtain regional LS longitudinal strain values (averaged basal, averaged mid, and averaged apical strain values). Regional CS was assessed considering strain values for the six basal, six mid and 4 apical segments of the LV. All segments were analyzed separately as antero-lateral, infero-lateral, inferior, infero-septal and antero-septal wall at basal, mid-ventricular, and apical level and successively averaged to obtain regional CS strain values (averaged basal, averaged mid, and averaged apical strain values) [14]. The reproducibility of T1 relaxation time, ECV measurement and CMR-FT analysis was assessed on a random sample of 10 patients, analyzed by 2 different operators, blinded to clinical data with more than 5 years’ experience in CMR examination analysis (Additional file 1).

### Holter analysis

All patients underwent 24-h Holter monitoring within 6 months from CMR. Isolated ventricular premature beats (PVC), couplets, bigeminal PVC and non-sustained ventricular tachycardia (NSVT) or sustained ventricular tachycardia (SVT) were reported. Patients were divided into 2 groups according to the results of the Holter monitoring: the MAD-cVA group in case of cVA occurrence and the MAD-noVA group in the absence of cVA. Complex ventricular arrhythmias (cVA) were defined according to the classification proposed by Essayagh et all. As the following: mild with PVCs above the median (> 5%) and/or with documented ventricular tachycardia (VT) runs no faster than 120 beats/min (34); moderate with VT runs of 120 to 179 beats/min; and as severe with VT > 180 beats/min and/or proven history of VT/ventricular fibrillation (VF)/aborted sudden cardiac death (SCD), indicating a need for an implantable cardioverter-defibrillator (ICD) [15]. The MAD-noVA group was defined as patients presenting with no/trivial VT and PVC frequency below median (< 5%).

### Statistical analysis

Normality of continuous variables was investigated with the Shapiro-Wilks test. Continuous variables are expressed as mean ± SD in case of a normal distribution or as median [interquartile range] in case of a non-normal distribution. Comparison of continuous variables between two groups were performed using the independent-sample Student’s t test in case of normal distribution or the Mann–Whitney test in case of non-normal distribution. One-way analysis of variance (ANOVA) was used to determine statistically significant differences in the presence of three or more independent groups normally distributed. The association between any categorical variable and outcome was analyzed using Fisher’s exact test. Correlation analysis was performed using Spearman’s correlation test. A whole series of univariate binary logistic models was used to estimate the potential relation between cVA (dependent variable) and every independent variable. A multivariate analysis was performed to estimate the potential relation between cVA (dependent variable) and a set of independent variables. We performed a univariate analysis considering all variables possibly correlated with higher incidence of arrhythmias (MAD length and MR regurgitant fraction, the presence of myocardial fibrosis evaluated on LGE and the presence of interstitial fibrosis evaluated on T1 mapping and the presence of myocardial deformation evaluated by CMR-FT). Multivariable analysis was performed in a stepwise fashion based on p model. In the iterative process of variable selection, covariates were removed from the model if they are non-significant and not a confounder. Significance was evaluated at the 0.1 alpha level. A Hosmer–Lemeshow goodness of fit (GOF) test was made in order to validate the multivariate model. Reproducibility analyses for the measurement of myocardial pre-contrast T1, ECV and strain parameters (GLS and GCS) were performed using Pearson’s correlation and Bland–Altman statistics. All reported p-values were obtained by the two-sided exact method at the 5% significance level. Data analysis was performed as of August 2021 by using the software package R (version 3.6.3, R Foundation for Statistical Computing, Vienna-A, http://www.R-project.org).

## Results

A total of 47 consecutive patients with MVP and MAD were identified. Five patients with complex congenital heart disease were excluded, leading to a final population of 42 patients. Holter monitoring analysis and history of aborted sudden cardiac death led to the identification of 23 patients (55%) in the MAD-cVA group and 19 patients (45%) in the MAD-noVA group.

### Baseline clinical and morphological characteristics

Mean age was 48.1 ± 15.4 years and similar across MVP patients vs control group (p = 0.198); 50% of the population were females. LV end-diastolic volume index was higher in patients with MVP compared with controls (100.7 ± 28.9 ml/m^2^ vs 61.2 ± 17.3 ml/m^2^, p = 0.003). No statistically significant differences in terms of LV end-diastolic volume index were found between MAD-noVA and MAD-cVA (98.0 ± 25.9 ml/m^2^ vs 98.4 ± 29.2 ml/m^2^, p = 0.421 respectively). No difference in terms of LVEF were found between patients in MAD-cVA and MAD-noVA (p = 0.833). MR severity grade was graded as mild in 7 patients (16%), moderate in 25 (60%) and severe in 10 (24%). MAD length was not different between MAD-noVA and MAD-cVA patients (10.3 ± 4. mm vs 9.2 ± 4.4 mm, p = 0.417).

No difference was observed among the 3 MR severity grades in terms of MAD length (7.1 [6.8–9.8] mm for mild MR vs 10.2 [8.8–13.4] mm for moderate MR vs 8.7 [4.3–13.2] mm for severe MR, p = 0.150). Baseline clinical and CMR characteristics of the overall study population are presented in Table 1. According to the classification provided by Essayagh et al. [15] 14 patients (60%) presented with mild cVA, 2 patients (8%) with moderate cVA and 7 patients (30%) presented with severe cVA, among them 6 patients (23%) were known for aborted SCD.

**Table 1:** Clinical and CMR characteristics of overall study population compared to control population

	Overall population (n = 42)	Control population (n = 20)	P value
Age (± SD)	48.1 ± 15.4	53.3 ± 22.5	0.745
Male gender (%)	21 (50%)	7 (35%)	0.004
BMI (± SD)	26.4 ± 7.1	24.2 ± 3.5	0.234
BSA (m^2^ ± SD)	1.8 ± 0.2	1.7 ± 0.3	0.412
EDV (ml ± SD)	**187 ± 55.9**	**118 ± 39.2**	**0.003**
EDVi (ml/m^2^ ± SD)	**101 ± 28.9**	**61.2 ± 17.3**	**0.003**
ESV (ml ± SD)	**82.1 ± 30.6**	**40.1 ± 21.5**	**0.004**
EF (% ± SD)	56.5 ± 7.9	60.2 ± 8.3	0.097
LV mass (g/m^2^ ± SD)	63.9 ± 15.4	62.1 ± 15.2	0.874
MAD length (mm ± SD)	9.3 ± 4.1	–	
MR		–	
– Mild	7 (16%)		
– Moderate	25 (60%)		
– Severe	10 (24%)		
Native T1 relaxation times (ms ± SD))			
– Anterior wall	1015 ± 91	1005 ± 101	0.154
– Anterolateral wall	1047 ± 70	994 ± 113	0.254
– Inferolateral wall	1083 ± 65	1001 ± 55	0.423
– Inferior wall	1101 ± 64	974 ± 64	**0.041**
– Inferoseptal wall	1074 ± 56	978 ± 85	0.078
– Anteroseptal wall	1063 ± 46	1011 ± 62	0.456
– Mean	1097 ± 45	1010 ± 26	**0.002**
ECV (% ± SD)			
– Anterior wall	0.27 ± 0.04	0.25 ± 0.04	**0.041**
– Anterolateral wall	0.29 ± 0.04	0.26 ± 0.04	**0.004**
– Inferolatera wall	0.30 ± 0.05	0.25 ± 0.04	**0.005**
– Inferior wall	0.30 ± 0.03	0.26 ± 0.04	**0.001**
– Inferoseptal wall	0.29 ± 0.04	0.26 ± 0.03	**0.004**
– Anteroseptal wall	0.30 ± 0.03	0.25 ± 0.04	**0.002**
– Mean	0.30 ± 0.03	0.25 ± 0.04	**0.022**
GLS (± SD)	− 25.8 ± 8.7	− 26.7 ± 9.4	0.256
LS basal (± SD)			
– Anterior wall	− 21.2 ± 4.5	− 22.8 ± 10.6	0.771
– Anterolateral wall	− 27.2 ± 10.5	− 25.6 ± 6.3	0.715
– Inferolateral wall	− 20.0 ± 8.4	− 24.6 ± 7.8	0.087
– Inferior wall	− 20.7 ± 7.5	− 24.4 ± 9.6	**0.047**
– Inferoseptal wall	− 17.6 ± 9.4	− 18.8 ± 9.5	0.457
– Anteroseptal wall	− 22.1 ± 4.5	− 22.5 ± 9.6	0.541
– Mean	− 22.1 ± 6.7	− 25.5 ± 11.4	0.521
LS mid–wall (± SD)			
– Anterior wall	− 21.8 ± 11.2	− 22.53 ± 7.6	0.771
– Anterolateral wall	− 22.7 ± 9.1	− 26.7 ± 11.6	0.715
– Inferolateral wall	− 25.7 ± 7.3	− 22.9 ± 9.7	0.087
– Inferior wall	− 21.9 ± 9.9	− 24.8 ± 7.5	0.087
– Inferoseptal wall	− 25.7 ± 8.3	− 27.1 ± 6.7	0.457
– Anteroseptal wall	− 22.7 ± 9.1	− 18.9 ± 9.1	0.541
– Mean	− 23.5 ± 8.7	− 22.1 ± 7.8	0.521
LS apical (± SD)			
– Anterior wall	− 28.7 ± 14.5	− 27.9 ± 9.6	0.715
– Septal wall	− 26.7 ± 11.4	− 33.2 ± 19.0	0.287
– Lateral wall	− 23.4 ± 8.1	− 21.0 ± 13.9	0.474
– Inferior wall	− 28.0 ± 12.6	− 34.8 ± 15.5	0.457
– Mean	− 25.6 ± 12.9	− 28.3 ± 18.0	0.541
CS basal (± SD)			
– Anterior wall	− 25.1 ± 4.1	− 26.7 ± 4.7	0.871
– Anterolateral wall	− 27.1 ± 5.1	− 24.1 ± 5.1	0.215
– Inferolateral wall	− 24.2 ± 5.8	− 27.1 ± 5.4	**0.451**
– Inferior wall	− 19.7 ± 5.4	− 27.6 ± 7.2	**0.021**
– Inferoseptal wall	− 23.4 ± 8.1	− 27.5 ± 5.5	0.457
– Anteroseptal wall	− 22.4 ± 7.0	− 24.5 ± 5.8	0.541
– Mean	− 24.2 ± 4.5	− 25.6 ± 4.5	0.521
CS mid-ventricular (± SD)			
– Anterior wall	− 20.8 ± 4.2	− 21.5 ± 5.6	0.871
– Anterolateral wall	− 21.2 ± 5.5	− 22.5 ± 6.7	0.215
– Inferolateral wall	− 22.9 ± 7.5	− 26.4 ± 7.7	0.048
– Inferior wall	− 16.1 ± 7.7	− 22.6 ± 3.6	0.021
– Inferoseptal wall	− 20.8 ± 6.1	− 21.5 ± 7.6	0.457
– Anteroseptal wall	− 21.5 ± 7.9	− 22.5 ± 6.8	0.541
– Mean	− 20.5 ± 4.5	− 21.5 ± 5.6	0.52
CS apical (± SD)			
– Anterior wall	− 28.6 ± 12.2	− 29.7 ± 11.5	0.401
– Septal wall	− 29.4 ± 21.0	− 30.5 ± 5.2	0.782
– Inferior wall	− 32.4 ± 13.7	− 28.7 ± 12.5	0.094
– Lateral wall	− 32.7 ± 13.1	− 35.6 ± 12.5	0.357
– Mean	− 28.1 ± 6.8	− 32.5 ± 11.5	0.541

Statistically significant values are highlighted in bold

*BMI* body mass index, *EDV* end-diastolic volume, *EDVi* end-diastolic volume indexed. *ESV* end-systolic volume, *EF* ejection fraction, *LV* left ventricle, *MAD* mitro-annular disjunction, *MR* mitral regurgitation, *ECV* extracellular volume fraction, *GLS* global longitudinal strain, *LS* longitudinal strain, *CS* circumferential strain

### Strain parameters

**Fig. 1:**
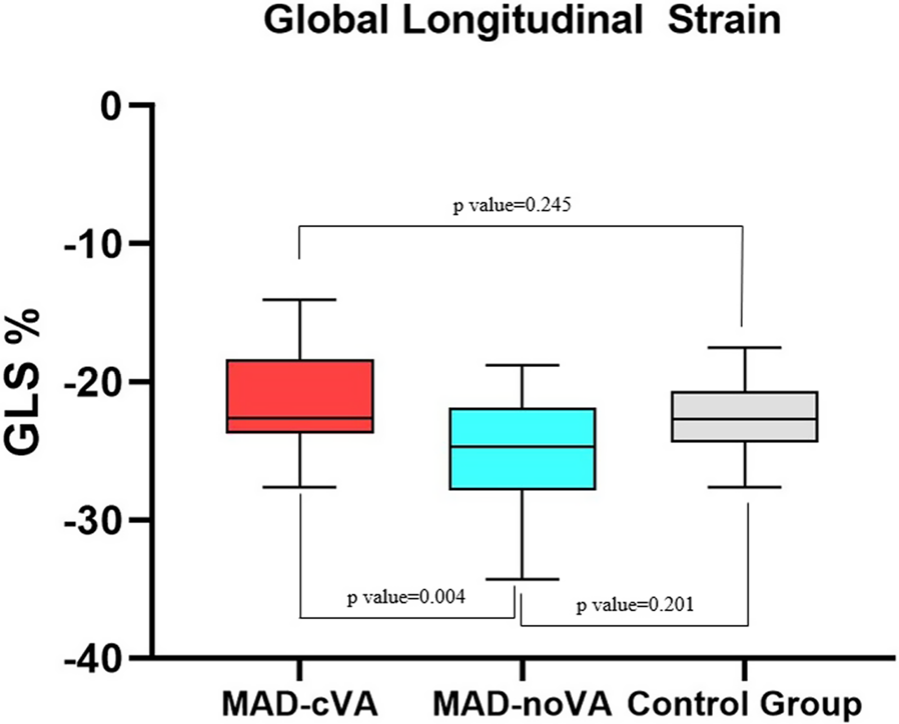
Box-plot representing GLS values for patients with MAD-cVA, MAD-noVA and control group. *GLS* Global Longitudinal Strain, *MAD* mitral annulus disjunction, *cVA* complex ventricular arrhythmias, *no-VA* no complex ventricular arrhythmias

**Fig. 2:**
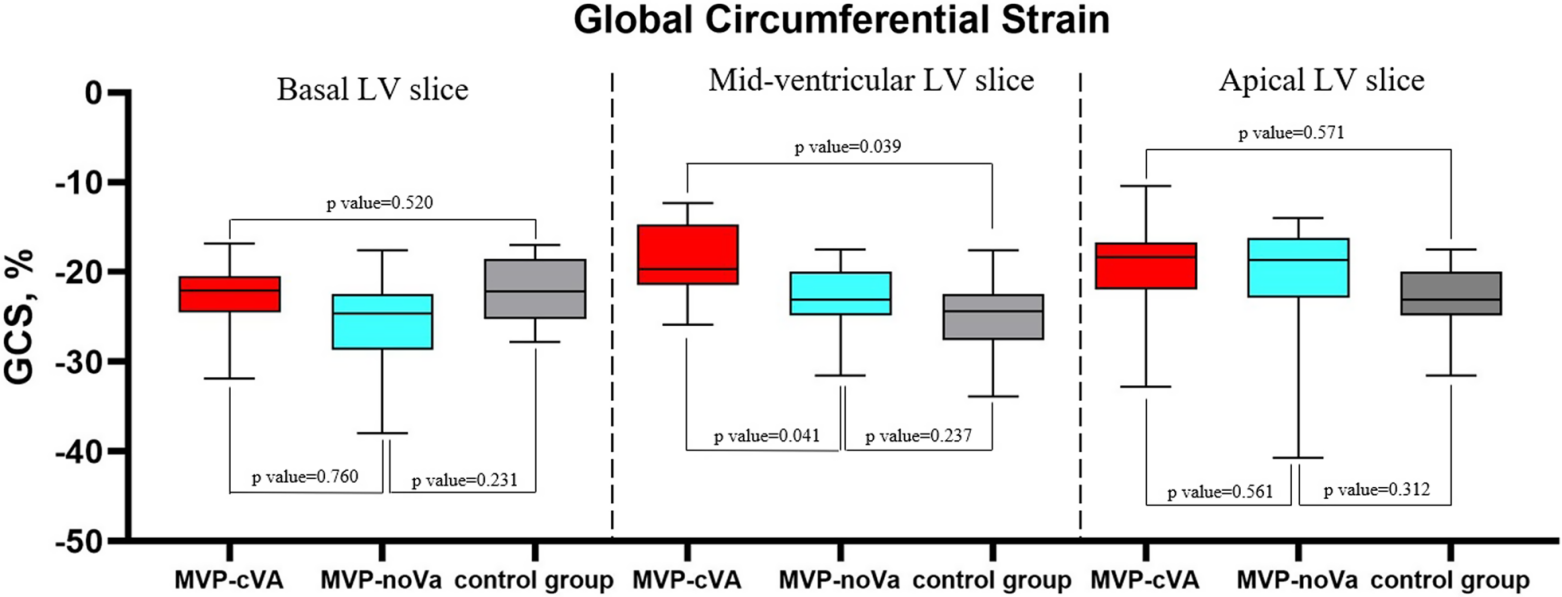
Box-plot representing GCS values at basal LV, mid-ventricular LV and apical LV level in MAD-cVA, MAD-noVA and control group. *GCS* global circumferential strain, *LV* left ventricle, *MAD* mitral annulus disjunction, *cVA* complex ventricular arrhythmias, *no-VA* no complex ventricular arrhythmias

No segments with poor tracking were present. No significant differences were found between patients with MVP and control group in terms of GLS (− 25.8% ± 8.7% vs − 26.7% ± 9.4%, p = 0.256). Similarly, GCS was not different between patients with MVP and control group at the basal (− 24.2% ± 4.5% vs − 25.6% ± 4.5%, p = 0.521), mid-ventricular (− 20.5% ± 4.5% vs − 21.5% ± 5.6%, p = 0.520) and apical levels (− 28.1% ± 6.8% vs − 32.5% ± 11.5%, p = 0.541). On the contrary, GLS was significantly reduced in MAD-cVA patients in comparison with MAD-noVA patients (− 18.2% ± 4.5% vs − 25.1% ± 3.1%, p = 0.004) as well as GCS at the mid-ventricular level (− 17.5% ± 4.7% vs − 21.6% ± 3.1%, p = 0.041). No difference was found in terms of GCS at the basal and apical levels (− 22.4% ± 4.5% vs − 24.7% ± 6.4%, p = 0.765 and − 30.8% ± 8.6% vs − 33.1% ± 5.4% p = 0.561, respectively) (Figs. 1 and 2).

**Fig. 3:**
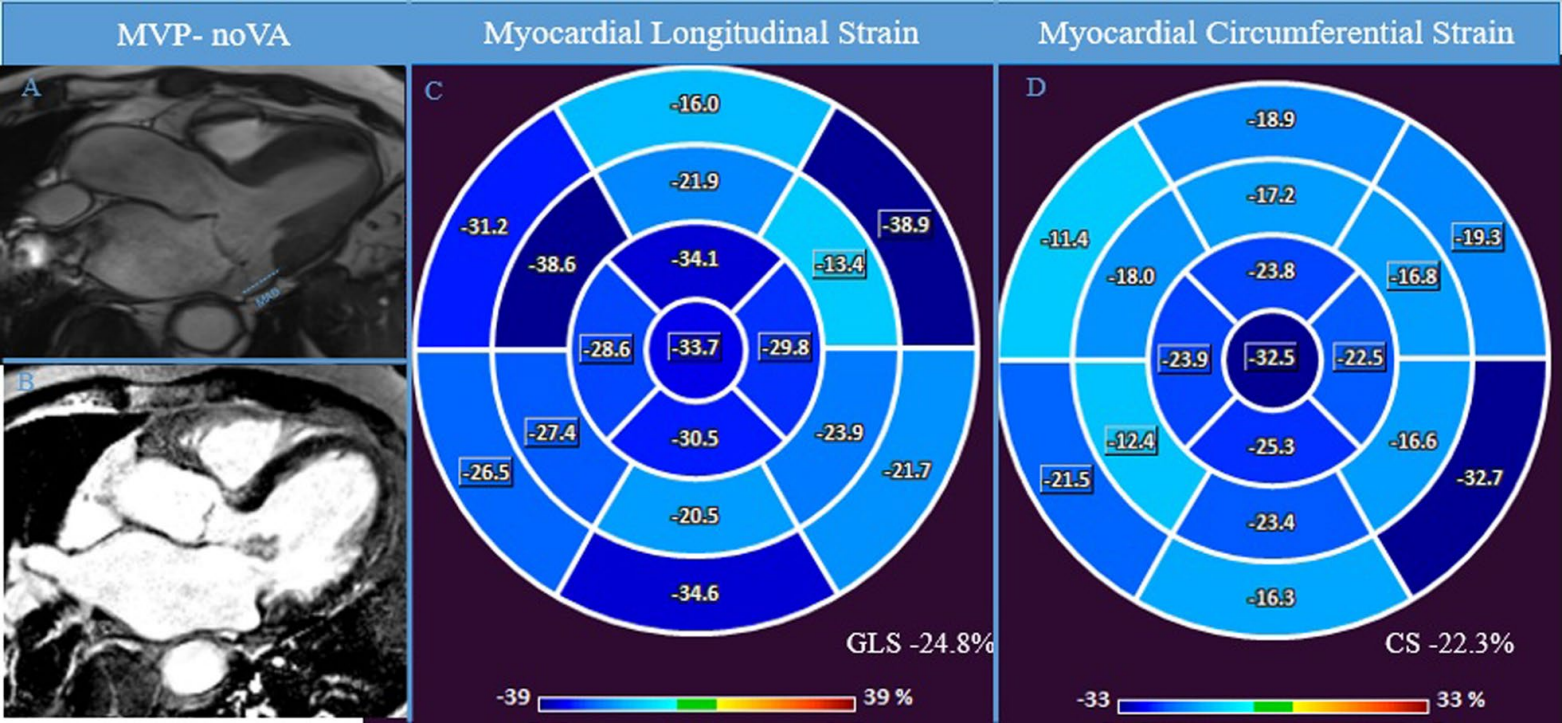
MAD-noVA. CMR examination of a patient known for known for bileaflet MVP with MAD (7.4 mm) without cVA at 24-h-Holter monitoring. Panel **A** shows 3 chamber view showing systolic MAD and the bileaflet MVP. Panel **B** shows the absence of macroscopic fibrosis in LGE and normal regional LS and GLS and GCS was finally evaluated in CMR-FT (Panel **C**-**D**). *CMR* cardiac magnetic resonance, *FT* feature-tracking, *GCS* global circumferential strain, *GLS* global longitudinal strain, *LGE* late gadolinium enhancement, *LS* longitudinal strain, *MVP* mitral valve prolapse, *MAD* mitral annulus disjunction

**Fig. 4:**
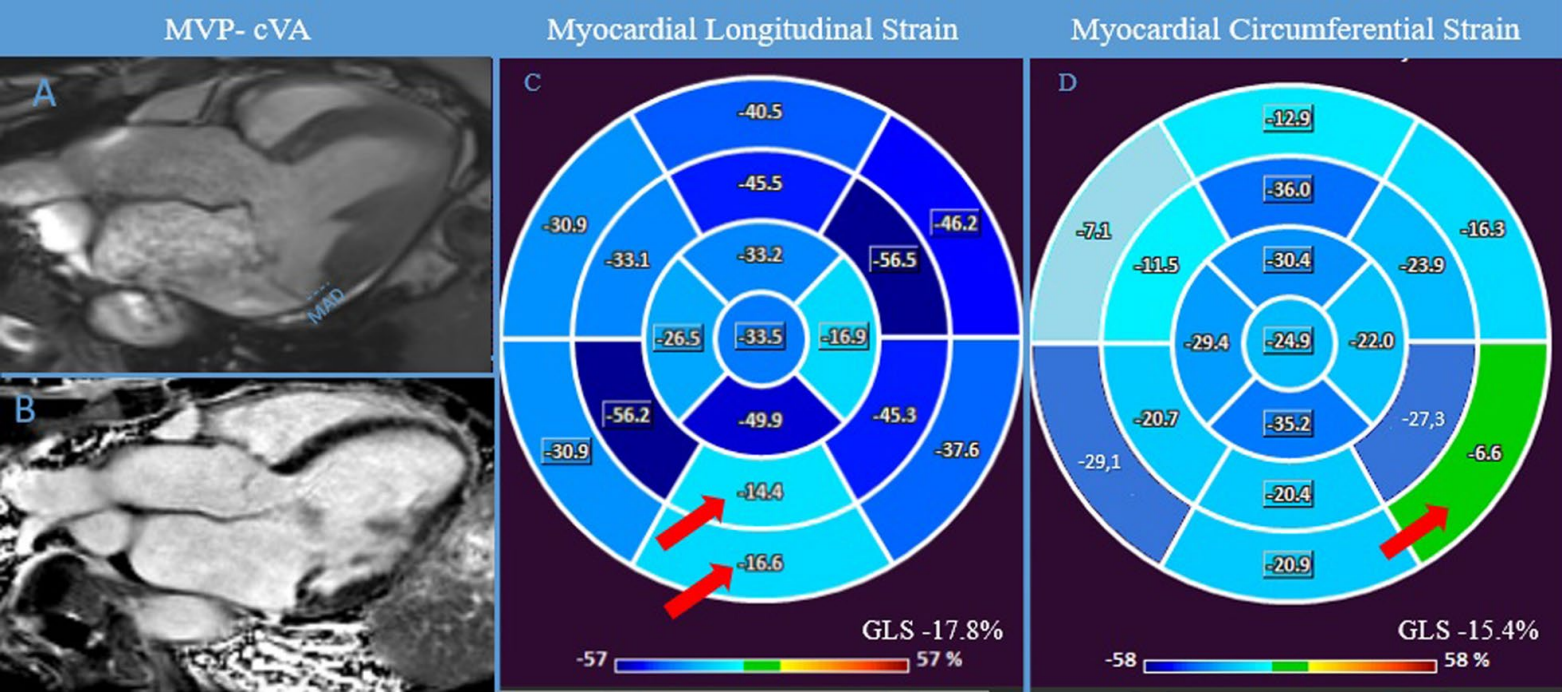
MAD-cVA. CMR examination of a patient known for bileaflet MVP with MAD (8.1 mm) with cVA at 24-h-Holter monitoring. Panel **A** shows 3 chamber view showing systolic MAD (blue lines) and the bileaflet MVP. Panel **B**, displays the presence of LGE in the inferolateral wall.  Abnormal GLS was found, with lower values of regional LS in the inferior wall (red arrow) (Panel **C**). Similarly, also GCS was reduced with lower values in the infero-lateral basal wall (red arrow) (Panel **D**). *CMR* cardiac magnetic resonance, *cVA* complex ventricular arrhythmias, *FT* feature-tracking, *GCS* global circumferential strain, *GLS* global longitudinal strain, *LGE* late gadolinium enhancement, *LS* longitudinal strain, *MAD* mitral annulus disjunction, *MVP* mitral valve prolapse

No significant differences were found between patients with MVP and control group in terms of regional strain (all p > 0.05), except for the regional LS of the basal inferior wall (− 20.7% ± 7.5% vs − 24.4% ± 9.6%, p = 0.047). Regional LS and CS measures are presented in Table 1. Regional LS was reduced in MAD-cVA patients when compared to MAD-noVA patients in the basal inferior wall (− 16.8% ± 7.9% vs − 23.1% ± 7.0%, p = 0.007). Regional CS was reduced in the basal infero-lateral wall (− 19.5% ± 7.6% vs − 27.4% ± 7.5%, p =  < 0.001), basal inferior wall (− 19.6% ± 6.2% vs − 26.2% ± 6.6%, p = 0.008) and in the mid ventricular infero-lateral wall (− 14.7% ± 8.2% vs − 18.9% ± 6.9%, p = 0.053) in MAD-cVA patients compared to MAD-noVA (Figs. 3 and 4).

Baseline clinical and CMR characteristics and strain parameters of MAD-noVA and MAD-cVA are reported in Table 2.

**Table 2:** Basal clinical and CMR characteristics of MVP cVA and MVP noVA

	MAD cVA (23 patient)	MAD noVA (19 patient)	P value
Age (± SD)	46.6 ± 13.0	49.6 ± 17.8	0.198
Male gender (%)	10 (43%)	11 (57%)	0.887
BMI (± SD)	26.4 ± 4.5	24.1 ± 4.7	0.612
BSA (m^2^ ± SD)	1.7 ± 0.2	1.8 ± 0.3	0.452
EDV (ml ± SD)	181.4 ± 48.2	184.3 ± 46.1	0.468
EDVi (ml/m^2^ ± SD)	98.0 ± 25.9	98.4 ± 29.2	0.421
ESV (ml ± SD)	79.6 ± 25.1	83.2 ± 34.2	0.537
EF (% ± SD)	55.9 ± 8.4	57.2 ± 6.8	0.833
LV mass (g/m^2^ ± SD)	62.3 ± 12.4	71.9 ± 14.2	0.486
MAD length (mm ± SD)	10.3 ± 4.5 [7.3–12.3]	9.2 ± 4.4 [6.7–11.5]	0.417
MR			
– Mild	3 (13%)	4 (21%)	
– Moderate	14 (61%)	11 (58%)	
– Severe	6 (26%)	4 (21%)	
LGE	18 (78%)	8 (42%)	0.0002
Native T1 mapping (ms (± SD)			
– Anterior wall	1021 ± 54	1011 ± 93	0.634
– Anterolateral wall	1052 ± 56	1051 ± 65	0.401
– Inferolateral wall	**1104 ± 63**	**1074 ± 63**	**0.026**
– Inferior wall	**1116 ± 58**	**1087 ± 58**	**0.015**
– Inferoseptal wall	1085 ± 59	1082 ± 48	0.167
– Anteroseptal wall	1062 ± 40	1054 ± 44	0.611
– Mean	**1083 ± 41**	**1044 ± 52**	**0.039**
ECV (% ± SD)			
– Anterior wall	0.27 ± 0.04	0.27 ± 0.04	0.336
– Anterolateral wall	0.29 ± 0.03	0.29 ± 0.05	0.464
– Inferolateral wall	0.31 ± 0.05	0.30 ± 0.04	0.221
– Inferior wall	0.31 ± 0.03	0.30 ± 0.02	0.307
– Inferoseptal wall	0.29 ± 0.03	0.29 ± 0.04	0.87
– Anteroseptal wall	0.28 ± 0.04	0.30 ± 0.03	0.235
– Mean	0.30 ± 0.04	0.29 ± 0.04	0.791
GLS (± SD)	**− 18.2 ± 4.6**	**− 25.1 ± 3.1**	**0.004**
LS basal (± SD)			
– Anterior wall	− 17.0 ± 8.2	− 17.9 ± 9.1	0.924
– Anterolateral wall	− 22.8 ± 8.3	− 22.1 ± 7.4	0.288
– Inferolateral wall	− 21.1 ± 11.0	− 24.9 ± 9.3	0.459
– Inferior wall	**− 16.8 ± 7.9**	**− 23.1 ± 7.0**	**0.007**
– Inferoseptal wall	− 18.6 ± 6.3	− 20.8 ± 9.8	0.245
– Anteroseptal wall	− 27.5 ± 13.1	− 24.3 ± 8.1	0.718
– Mean	− 24.3 ± 8.1	− 22.9 ± 9.1	0.341
LS mid-wall (± SD)			
– Anterior wall	− 25.8 ± 10.7	− 26.2 ± 8.3	0.719
– Anterolateral wall	− 24.2 ± 7.3	− 29.7 ± 7.6	0.63
– Inferolateral wall	− 30.8 ± 7.6	− 26.5 ± 6.3	0.071
– Inferior wall	− 29.5 ± 6.8	− 28.6 ± 6.1	0.76
– Inferoseptal wall	− 24.2 ± 7.3	− 25.3 ± 7.2	0.994
– Anteroseptal wall	− 22.4 ± 8.2	− 21.0 ± 7.6	0.183
– Mean	− 24.5 ± 7.2	− 24.5 ± 8.4	0.147
LS apical (± SD)			
– Anterior wall	− 27.6 ± 13.9	− 28.0 ± 11.2	0.679
– Septal wall	− 22.3 ± 8.0	− 29.7 ± 12.6	0.154
– Inferior wall	− 27.0 ± 12.8	− 29.7 ± 12.6	0.28
– Lateral wall	− 26.9 ± 7.0	− 28.0 ± 7.0	0.253
– Mean	− 29.0 ± 10.9	− 34.6 ± 11.0	0.131
CS basal (± SD)			
– Anterior wall	− 24.5 ± 5.4	− 26.1 ± 6.5	0.315
– Anterolateral wall	− 25.8 ± 5.5	− 25.6 ± 5.5	0.887
– Inferolateral wall	**− 19.5 ± 7.6**	**− 27.4 ± 7.5**	**0.0001**
– Inferior wall	**− 19.6 ± 6.2**	**− 26.2 ± 6.6**	**0.0078**
– Inferoseptal wall	− 23.1 ± 5.0	− 24.4 ± 5.9	0.927
– Anteroseptal wall	− 23.1 ± 5.0	− 26.1 ± 7.4	0.713
– Mean	− 22.4 ± 4.5	− 24.7 ± 6.4	0.765
CS midventricular (± SD)			
– Anterior wall	− 20.7 ± 4.8	− 20.3 ± 3.4	0.359
– Anterolateral wall	− 22.1 ± 6.7	− 20.3 ± 4.3	0.21
– Inferolateral wall	− **14.7 ± 8.2**	− **18.9 ± 6.9**	**0.053**
– Inferior wall	− 23.3 ± 8.1	− 24.3 ± 7.5	0.526
– Inferoseptal wall	− 22.1 ± 6.9	− 19.9 ± 5.5	0.273
– Anteroseptal wall	− 23.2 ± 5.2	− 19.7 ± 9.9	0.245
– Mean	**− 17.5 ± 4.7**	**− 21.6 ± 3.1**	**0.041**
CS apical (± SD)			
– Anterior wall	− 32.5 ± 16.1	− 24.8 ± 7.2	0.579
– Septal	− 26.3 ± 32.0	− 31.9 ± 8.0	0.097
– Inferior	− 38.6 ± 12.4	− 35.0 ± 15.3	0.715
– Lateral	− 34.1 ± 20.2	− 31.6 ± 4.4	0.614
– Mean	− 30.8 ± 8.6	− 33.1 ± 5.4	0.561

Statistically significant values are highlighted in bold

*BMI* body mass index, *cVA* complex ventricular arrythmias, *EDV* end-diastolic volume, *EDVi* end-diastolic volume indexed. *ESV* end-systolic volume, *EF* ejection fraction, *LV* left ventricle, *MAD* mitral annular disjunction, *MR* mitral regurgitation, *noVA* no complex ventricular arrhythmias, *ECV* extracellular volume, *GLS* global longitudinal strain, *LS* longitudinal strain, *CS* circumferential strain

### Tissue characterization

The proportion of patients with LGE was higher in the MAD-cVA group in comparison with the MAD-noVA group (78% vs 42%, p = 0.002). The LGE extension was of 3 [2–4] in the group with cVA vs 0 [0–1] in the group without cVA (p = 0.001). LGE was most frequently detected in the basal to mid-ventricular inferior and inferolateral walls (i.e. in the vicinity of the posterior mitral leaflet attachments) as opposed to the anterior and anterolateral walls (52% vs 11%, p = 0.001) and the anteroseptal and inferoseptal walls (58% vs 11%, p = 0.002).

Patients with MVP had higher mean pre-contrast T1 relaxation times compared with controls (1097 ± 45 ms vs 1010 ± 26 ms, p = 0.002) and higher ECV values (0.30 ± 0.03% vs 0.25 ± 0.04%, p = 0.022). Patients with cVA had higher pre contrast T1 relaxation times of the inferior basal wall (1116 ± 58 ms vs 1087 ± 58 ms, p = 0.015) and inferolateral basal wall levels (1104 ± 63 ms vs 1074 ± 63 ms, p = 0.026) when compared to noVA patients, while no difference was found in terms of ECV values (0.30 ± 0.04 vs 0.29 ± 0.04, p value 0.791). Native T1 and ECV values are presented in Table 2.

### Association between tissue characterization, strain parameters and arrhythmia

Seven patients presented with polymorphic arrhythmias, all of them in the MAD-cVA group. Five patients with polymorphic arrhythmias presented > 5%.

Univariate logistic regression analysis identified the following covariates as unadjusted predictors of the incidence of cVA: presence of LGE (OR: 9.52 [2.28–39.7] p = 0.002), the number of segments with LGE (OR: 1.78 [1.21–2.63], p = 0.004), GLS (OR: 1.58 [1.21–2.07], p < 0.001), regional CS in the basal infero-lateral wall (OR: 9.52 [2.28–39.7], p = 0.001) regional CS in the infero-lateral mid ventricular wall (OR: 1.41 [1.16–1.72], p = 0.006), regional LS in the basal and mid-ventricular infero-lateral wall (OR: 1.11 [1.00–1.23], p = 0.047 and OR: 1.62 [1.22–2.13], p < 0.001 respectively), pre contrast T1 relaxation times and ECV in the basal inferolateral wall (OR: 1.01 [1.00–1.02], p = 0.038 and OR: 1.12 [1.22–1.98], p = 0.047, respectively). MAD length and mitral regurgitant fraction were not predictors of cVA.

We performed a multivariate analysis including all the possible parameters that may be connected to cVA such as the degree of MR, the presence and extent of LGE, the presence of interstitial fibrosis evaluated in native T1 mapping end ECV and myocardial deformation parameters. In our cohort, the presence of LGE [OR: 8.5 (IC95%: 1.9–38.2; p = 0.008)], GLS [OR:1.56 (IC95%: 1.45–2.47; p = 0.007)] and the regional LS in the basal infero-lateral wall [OR: 1.62 (IC95%: 1.22–2.13; p < 0.001)] were independent prognostic factors in multivariate analysis. The ROC curve analysis showed an area under the curve of 0.92 (IC95%: 0.824–1) and the Hosmer and Lemeshow GOF test confirmed the validity of the model. Logistic univariate and multivariate analyses are presented in Table 3.

**Table 3:** univariate and multivariate analysis of predictors of c-VA

Univariate logistic analysis of predictors of cVA				Multivariate analysis stepwise based on p model**		
	OR	95% CI	P value	OR	95% CI	P value
MR regurgitant fraction (%)	89.40	0.66–1.22	0.0723	–	–	–
LGE	9.52	2.28–39.7	0. 002	8.5	1.9–38.2	0.008
Number of LGE segments	1.78	1.21–2.63	0.004	**–**	**–**	**–**
MAD length	1.18	0.97–1.44	0.087	–	–	–
Mean native T1 relaxation time (ms)	1.01	0.97–1.03	0.130	–	–	–
Native T1 relaxation time of inferolateral wall (ms)	1.01	1.00–1.02	0.038	**–**	**–**	**–**
Native T1 relaxation time of inferior wall (ms)	1.01	0.99–1.02	0.256	–	–	–
Mean ECV	1.24	0.08–1.87	0.222	–	–	–
ECV inferior wall	1.11	0.01–3.64	0.127	–	–	–
ECV inferolateral wall	1.12	1.22–1.98	0.047	**–**	**–**	**–**
GLS (%)	1.58	1.21–2.07	0.0009	1.6	1.45–2.47	0.007
LS basal Inferior wall (%)	1.11	1.00–1.23	0.545	1.6	1.22–2.13	0.0007
LS midventricular Inferior wall (%)	1.05	0.97–1.13	0.185	–	–	–
LS basal inferolateral wall (%)	1.11	1.00–1.23	0.047	**–**	**–**	**–**
LS midventricular inferolateral wall (%)	1.62	1.22–2.13	0.0007	**–**	**–**	**–**
CS (%)	1.13	0.98–1.29	0.077	–	–	–
CS basal inferolateral wall (%)	9.52	2.28–39.70	0. 001	**–**	**–**	**–**
CS mid-ventricular inferolateral wall (%)	1.41	1.16–1.72	0.0005	**–**	**–**	**–**
CS basal inferior wall (%)	1.00	1.000–1.010	0.0675	–	–	–
CS midventricular inferior wall (%)	3.58	0.8760–14.60	0.0759	2.1	1.87–3.17	0.006

*MR* mitral regurgitation, *LGE* late gadolinium enhancement, *MAD* mitral annular disjunction, *ECV* extracellular volume fraction, *GLS* global longitudinal strain, *LS* longitudinal strain, *GCS* global circumferential strain, *CS* circumferential strain

^*^Hosmer and Lemeshow goodness of fit (GOF) test: X-squared = 7.5543, df = 8, p-value = 0.4446. Percentage correct 92.68%

## Discussion

The main findings of the present study are:

1. the presence and extent of macroscopic fibrosis detected by LGE was associated with a higher incidence of cVA;
2. patients with cVA had a higher degree of ventricular remodeling highlighted by higher values of native T1 relaxation times and ECV;
3. cVA was associated with alterations in deformation parameters evaluated by CMR-FT.

Although a benign condition in the vast majority of cases, MVP has been linked to cVA and SCD in a subgroup of patients, justifying efforts to identify risk factors of ventricular arrhythmia [4, 6]. Among these factors, a growing interest has emerged on the presence of macroscopic fibrosis detected in LGE sequences and MAD, both highlighted as “high risk features” for cVA [6, 7] (Additional file 1: Fig. S2).

Kitkungvan et al. demonstrated that the prevalence of LV LGE in MVP patients is higher compared to non-MVP patients, suggesting that MVP fibrosis has a unique pathophysiology beyond volume overload [16]. Moreover, Constant Dit Beaufils et al. showed that LV myocardial fibrosis identified with LGE is common in patients with MVP, is associated with ventricular arrhythmias and independently related to cardiovascular events [17].

The concept of MAD was first introduced by Bharati et al. in 1981 [18], and since then, several studies have investigated its role in arrhythmogenesis [8, 9, 19]. The largest cohort published so far considering the prognosis of MAD has shown that its presence was independently associated with long-term incidence of clinical arrhythmic events, but not to an increased risk of mortality [15]. Of note, in our population of patients with MVP and MAD, cVA were present only in half of cases and MAD length failed to demonstrate an association with cVA both in univariate and multivariate analysis. It must be noted that following anatomical findings of Angelini et al. [17], Toh et al. [18] and Zugwitz et al. [19] shows that MAD appears to be a common feature present in normal adult heart. Based on these findings, the hypothesis of two patterns of MAD has recently been evocated [21]. A “pseudo MAD”, in which MAD is only detected in systole as the juxtaposition of the belly of the billowing posterior leaflet on the adjacent left atrial wall, giving the illusion that a disjunction is present, but a normal attachment of the leaflet can be observed in the diastolic phase, and a “true MAD”, when the disjunction can be seen in both systole and diastole and it is linked to an abnormal attachment of the leaflet in the atrial wall [21]. Surely, further studies to validate this hypothesis and to correlate with the risk of arrhythmias are needed, but this aspect brings back to the fore the need for a deepened understanding of myocardial composition in patients with MVP [20]. The presence of myocardial macroscopic fibrosis detected on LGE sequences in the inferior and infero-lateral wall as a possible key feature in genesis of arrhythmia in MVP patients was first described by Basso et al. [8] and confirmed thereafter in other studies.

Our results are in line with these findings as 78% of patients with known cVA or aborted SCD had macroscopic fibrosis detected in LGE sequences, while only 42% of patients in the no-cVA group were LGE positive, further supporting the importance of fibrosis in the stratification of the arrhythmic risk.

In addition to focal fibrosis, interstitial fibrosis assessed by T1 mapping may contribute to arrhythmogenesis in patients with MVP Bui et al. suggested that patients with MVP may present with higher levels of interstitial fibrosis as evaluated with native T1 mapping [21]. The authors demonstrated that diffuse fibrosis can contribute to complex ventricular arrhythmias even in absence of focal fibrosis [21].

This aspect has also recently been validated in a series [10] that highlighted how all patients with MVP and MAD presented with higher ECV levels and presented a higher risk of out-of-hospital cardiac arrest compared to MVP without MAD, compatible with the presence of interstitial fibrosis.

In our population, MVP and MAD also correlates with the presence of diffuse interstitial fibrosis with higher values of native T1 relaxation times and ECV compared to controls. However, the difference between mean ECV values in MAD-cVA and MAD-noVA was not statistically significant, even though mean ECV values showed a higher trend in MAD-cVA compared to MAD-noVA. On the contrary, higher values of native T1 relaxation times in the inferior and infero-lateral wall were detected in MAD-cVA.

Strain deformation parameters are known to be superior in the evaluation of EF in predicting LV dysfunction and major adverse cardiac events in different cardiac conditions [23].

Using 2D speckle tracking echocardiography (STE), Huttin et al. showed an abnormal strain pattern in MVP patients compared with controls, with pathological pre-systolic shortening and systolic, late systolic and post-systolic strain [22]. Moreover, STE derived mechanical dispersion in MVP may help to identify patients at higher risk for ventricular arrhythmias [23].

However, echocardiography is limited in the presence of inadequate acoustic windows. On the other hand, CMR-FT is obtained by post-processing conventional cine sequences which are characterized by a high natural contrast difference between the myocardium and the blood cavity. This novel technique which allows quantitative analysis of myocardial deformation, is based on optical flow methods [24].

CMR-FT is not necessarily comparable to speckle tracking echocardiography. In particular, because cine SSFP images show relatively homogeneous gray levels, CMR FT does not seem to be able to distinguish intramyocardial features as STE does. Indeed, CMR FT involves the detection of anatomical characteristics located along the myocardial boundaries within the image. It then proceeds to pinpoint regions of interest in proximity to these features and subsequently tracks them throughout the cardiac cycle by comparing corresponding regions in successive images [25]. Furthermore, cine CMR images have substantially lower spatial and temporal resolution than STE [24, 25]. These differences might explain why, unlike findings from studies using speckle tracking echocardiography [26], but consistent with prior findings using CMR-FT [11, 27] we did not observe differences in GLS between MVP patients and controls, even in the presence of significant mitral regurgitation.

Futhermore, we evaluated regional LS and CS at basal, mid-ventricular and apical level, demonstrating that regional changes in CMR-FT parameters in the inferior and inferolateral wall are also associated with cVA.

Taken together, the alterations in deformation, myocardial parameters and T1 mapping highlight the presence of ventricular remodeling that goes beyond the mere evidence of macroscopic fibrosis detected by LGE [23]. It can be speculated that the presence of interstitial fibrosis can cause subtle ventricular changes leading to the alterations of deformation parameters detected by CMR-FT in the inferior and inferolateral wall which would be the *primum movens* of macroscopic fibrosis that can be later detected in LGE sequences. However, the clinical role and the potential therapeutic target of interstitial fibrosis in patients with MVP still need further investigation in larger longitudinal studies.

Finally, the logistic univariate regression analysis suggests an important role for myocardial composition and deformation in the genesis of complex arrhythmias, since we found as stronger predictor of cVA the presence of macroscopic fibrosis detected in LGE as well as higher value of GLS and regional LS in the basal inferior wall. On the contrary, MAD length and MR severity failed to predict the incidence of cVA in our population in both univariate and multivariate analysis. Surely, these interesting results are limited by the number of patients and a more extensive analysis in a larger population on the role of MAD in arrhythmogenesis of patients with MVP is needed.

### Limitations

This study presents several limitations. Firstly, it is an observational study on a relatively limited number of patients. Moreover, we studied a highly selected population of patients with concomitant MVP and MAD that underwent ECG Holter monitoring. As a consequence, a selection bias may be present, and the multivariate analysis can be affected by model overfitting. Larger, prospective studies are required to confirm our data. However, although the combination between MVP and MAD is relatively rare in the general population, it appears particularly relevant from a clinical point of view, as it identifies a subgroup of patients at higher risk of arrhythmic events [28]. In addition, our study is the largest on the role of CMR-FT and tissue characterization in patients with bileaflet MVP and MAD.

Secondly, we derived information from ventricular arrhythmias on standard 3-lead Holter; consequently, information about the location of PVC is unavailable. Moreover, given the limited number of patients, no specific conclusion can be derived on the polymorphic or monomorphic nature of PVCs and their association with CMR-FT parameters and outcome. Finally, although high resolution bright blood LGE CMR were used and all images were interpreted by expert operators, routine evaluation of the presence of fibrosis in the papillary muscles area could be challenging and was not part of our analysis.

## Conclusion

In patients with bileaflet MVP and MAD, CMR identifies markers of fibrosis and subtle modification of myocardial deformation indexes which are associated with complex ventricular arrhythmias. LGE, T1 mapping and CMR-FT may represent potential tools to help the prognostic stratification of MVP patients at risk of SCD.

## Supplementary Information

**Additional file 1: Figure S1.** steady-state-free precessioncine image of a 3-chamber view, the presence on MADin highlighted with a red arrow. *MAD: mitral-annular disjunction***. Figure S2. **Considering the literature CMR evaluation in patients with suspicion of arrhythmic MVP includes the detection of MAD and curling in cine images, the presence of macroscopic fibrosis in LGE and the quantification of mitral regurgitation in 2D-phase contrast sequences of 4D flow, if available. The presence of interstitial fibrosisas well as the role of myocardial deformation by CMR feature tracking as possible marker of risk stratification are currently being evaluated.

## Data Availability

The datasets used and analyzed during the current study are available from the corresponding author on reasonable request.

## Abbreviations

cVA: Complex ventricular arrhythmias
CMR: Cardiovascular magnetic resonance
CS: Circumferential strain
EF: Ejection fraction
ECV: Extracellular volume
FT: Feature tracking
ICD: Implantable cardioverter-defibrillator
GLS: Global longitudinal strain
GCS: Global circumferential strain
LGE: Late gadolinium enhancement
LS: Longitudinal strain
LV: Left ventricle
MAD: Mitral annular disjunction
MR: Mitral regurgitation
MVP: Mitral valve prolapse
no-VA: No complex ventricular arrhythmias
NSVT: Non-sustained ventricular tachycardia
PVC: Isolated ventricular premature beats
SCD: Sudden cardiac death
STE: Speckle tracking echocardiography
SVT: Sustained ventricular tachycardia
VA: Ventricular arrhythmia
VF: Ventricular fibrillation
VT: Ventricular tachycardia
