# Supplementary material for: Additional value of cardiac magnetic resonance feature tracking parameters for the evaluation of the arrhythmic risk in patients with mitral valve prolapse
Source: J Cardiovasc Magn Reson. 2023 Jun 15;25:32. doi: 10.1186/s12968-023-00944-x (PMC10268415; doi:10.1186/s12968-023-00944-x)
Supplement: Supplementary file 1 — Additional file 1: Figure S1. steady-state-free precessioncine image of a 3-chamber view, the presence on MADin highlighted with a red arrow. MAD: mitral-annular disjunction. Figure S2. Considering the literature CMR evaluation in patients with suspicion of arrhythmic MVP includes the detection of MAD and curling in cine images, the presence of macroscopic fibrosis in LGE and the quantification of mitral regurgitation in 2D-phase contrast sequences of 4D flow, if available. The presence of interstitial fibrosisas well as the role of myocardial deformation by CMR feature tracking as possible marker of risk stratification are currently being evaluated. [file 12968_2023_944_MOESM1_ESM.docx]

**Additional Materials:**

**Intra-observer and Inter-observer variability:**

Intra-observer (r = 0.61, p = 0.004/Mean difference 4% − 95% levels of agreement—2 to 8 %) and inter-observer (r = 0.93, p < 0.001/Mean difference 5 % − 95% levels of agreement—4 to 7%) reproducibility for GCS was good. Both intra-observer (r = 0.99, p < 0.001/Mean difference 3 %–95% levels of agreement − 1% to 7% ms) and inter-observer (r = 0.99, p < 0.001/Mean difference 2% ms − 95% levels of agreement − 2% to 4%) reproducibility were excellent for GLS.

Intra-observer (r = 0.61, p = 0.004/Mean difference 9 ms − 95% levels of agreement—70 to 89 ms) and inter-observer (r = 0.93, p < 0.001/Mean difference 11 ms − 95% levels of agreement—25 to 46 ms) reproducibility for pre-contrast T1 relaxation time was good. Both intra-observer (r = 0.99, p < 0.001/Mean difference 0–95% levels of agreement − 0.3% to 0.4% ms) and inter-observer (r = 0.99, p < 0.001/Mean difference 0% ms − 95% levels of agreement − 0.4% to 0.4%) reproducibility were excellent for ECV calculation.


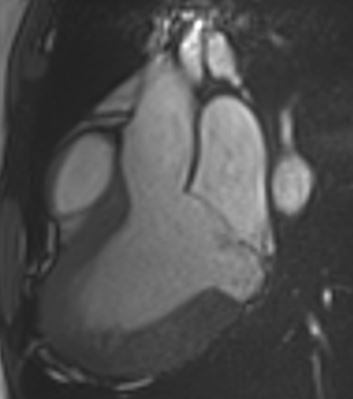


Fi

**Figure S1:** steady-state-free precession (SSFP) cine image of a 3-chamber view, the presence on MAD (in white) in highlighted with a red arrow. *MAD: mitral-annular disjunction*


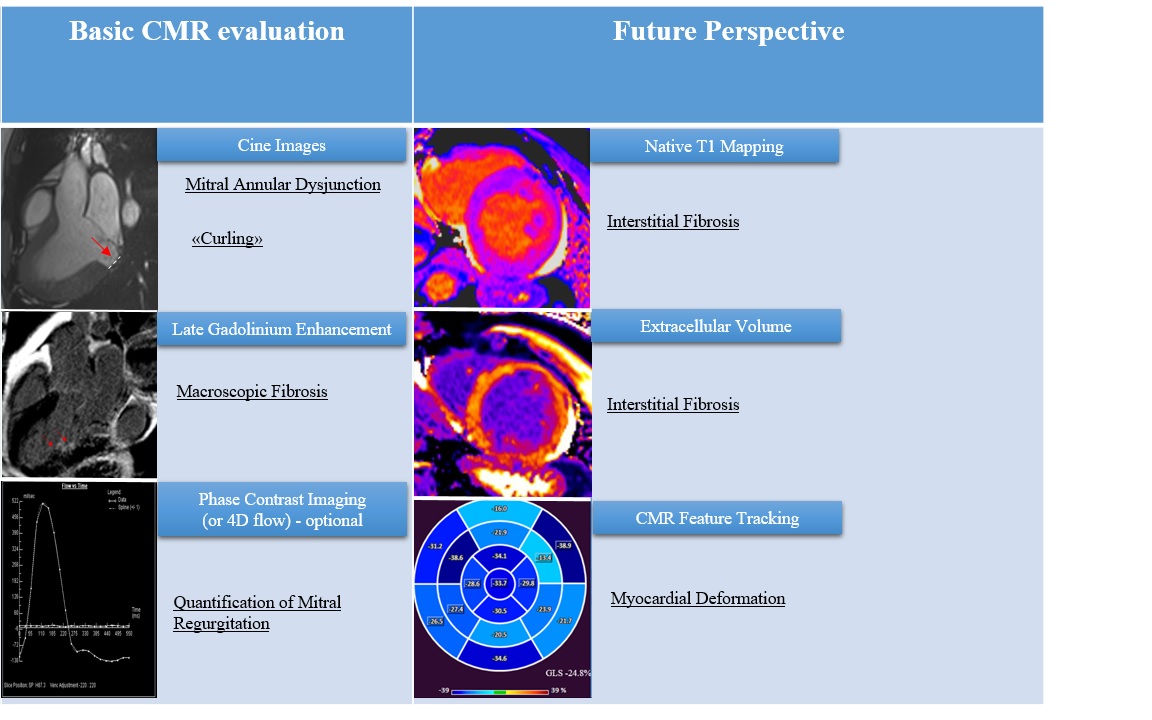


**Figure S2:** Considering the literature CMR evaluation in patients with suspicion of arrhythmic MVP includes the detection of MAD and curling in cine images, the presence of macroscopic fibrosis in LGE and the quantification of mitral regurgitation in 2D-phase contrast sequences of 4D flow, if available. The presence of interstitial fibrosis (evaluated in native T1 mapping or by calculating the extracellular volume) as well as the role of myocardial deformation by CMR feature tracking as possible marker of risk stratification are currently being evaluated.
